# Evaluation of mastalgia in patients presented to the breast clinic in Duhok city, Iraq: Cross sectional study

**DOI:** 10.1016/j.amsu.2020.02.012

**Published:** 2020-03-10

**Authors:** Ayad Ahmad Mohammed

**Affiliations:** Department of Surgery, College of Medicine, University of Duhok, Nakhoshkhana Road, 8 AM-1014, Duhok, Kurdistan Region, Iraq

**Keywords:** Mastalgia, Breast pain, Breast, Tamoxifen, Danazol

## Abstract

**Introduction:**

Mastalgia is a common condition that may affect up to two-thirds of patients during the reproductive period. It can be divided into cyclical and noncyclical. It is mostly due to benign causes, but breast cancer should be excluded. It may be associated with a high level of stress and anxiety. Patients need to be assessed fully, breast and general examinations, and investigations such as breast imaging and hormonal assay.

**Patients and methods:**

This is a prospective study that was done in the breast clinic in the period between February 2019 and July 2019. A total number of 445 patients with mastalgia included were in the study. Patients were examined and evaluated using various imaging modalities.

**Results:**

The mean age of the patients was 34 years. Mastalgia was present in 54.2% of our patients, in about 70.1% of our patients it was noncyclical mastalgia. Mastalgia was positively correlated with smoking, oral contraceptive pills, and positive family history of breast cancer (p values: 0.000, 0.009, and 0.000) respectively with no correlation with other factors. The type of pain was less in women having early first pregnancy and with the site of the pain showed a positive correlation with the type of pain, (p values: 0.001 and 0.000) respectively.

**Conclusion:**

Mastalgia is a common complaint which may affect most females. It is caused by benign breast disorders in the majority of patients. A systematic approach must be followed for the management of mastalgia. Reassurance, regular exercise, and local analgesics may be very effective initial measures. In severe, intractable cases, hormonal therapy may be used.

## Highlights

• Mastalgia is a common condition that may affect up to two-thirds.
• It can be divided into cyclical and noncyclical.
• It is mostly due to benign causes, but breast cancer should be excluded.
• Patients need full assessment by examination, investigations, imaging and hormonal assessments.

## 1 Introduction

Mastalgia or sometimes called mastodynia is a common condition that may present to the breast clinics or the family doctors.it may affect up to two-thirds of patients during their lifetime during the reproductive period. It is typically felt in the central part and ranges from tension, discomfort, or real pain. It can be divided into cyclical and noncyclical pain, depending on whether the breast pain is occurring in the premenstrual period or not; it usually has a chronic course. This condition is usually due to benign causes, but breast cancer should be excluded because it is the main concern in the majority of women, although it could be associated with premenstrual syndrome, anxiety disorders. Pain in extramammary sources needs to be also excluded like musculoskeletal pain, especially Tietz syndrome or referred pain [[1], [2], [3], [4]].

When the pain is severe and chronic, it affects the women's daily activities and may cause have a major impact on the mode and the work activities [4].

Mastalgia has been linked to a variety of conditions such as high level of stress and anxiety, depression, chronic myalgia, irritable bowel syndrome, chronic pelvic pain, and some psychiatric disorders which may suggest a psychosomatic base of this type of pain in some patients. Most authors agree that mastalgia has multifactorial pathogenesis [4,5].

It can be associated with a premenstrual syndrome, which may involve breast pain only or may be a state of generalized body pain and sensation of tension. The cause of the cyclical mastalgia is poorly understood, but it may be due to hormonal effects on the breast tissue or due to a systemic state of water and salts retention in the body [6].

Patients presented with mastalgia need to be assessed fully, including complete personal and family history, breast and general examinations, and the patient may need some investigations such as breast imaging and some of the hormonal assessments [7].

Mastalgia is variable in degrees of severity, in some patients it may cause some degree of discomfort or anxiety which mandates performing repeated investigations, and in the other group. **It** may be severe that disturb the lifestyle of the patients [7].

The most important step in the management after exclusion of cancer is reassurance [2,8,9].

Various treatment modalities have been tried for the effective treatment of mastalgia depending on the severity and the chronicity of this condition. Reassurance and work up that exclude cancer are the most important initial workups. Medical treatment has been tried in the form of analgesics, which could be used locally or systemically, which proved to be effective in mild to moderate forms of the complaint. Hormonal treatments like tamoxifen, danazol, and bromocriptine are also used in cases of severe mastalgia and when the first line medical treatment fails [4,10].

The work of this article has been reported in line with the STROCSS criteria [11].

**Aim and objectives:** To determine the associated factors that may be correlated with mastalgia.

## 2 Patients and methods

### 2.1 Study design and patients

This is a prospective study that was done in the breast clinic in the period between February 2019 and July 2019, we included 445 sequential patients who visited the breast clinic and were complaining from mastalgia.

### 2.2 Inclusions and exclusion criteria

All patients who presented to the surgical unit and complained from breast pain were included in this study, patients complained from other complains other than mastalgia and patients who refused to be included in this study were excluded from this study.

### 2.3 Diagnosis and measures

Patients presented with mastalgia included in the study, detailed information were taken from, and data recorded using a specially designed questionnaire. Patients underwent breast examination to detect the site of tenderness, lump or lumpiness, any associated nipple retraction or pain. Some patients were evaluated using various imaging modalities such as ultrasound, mammography, and MRI of the breast, and some patients sent for hormonal levels.

An informed consent was taken from all the participants to be included in this study.

No specific pre-intervention considerations were undertaken.

In accordance to the World Medical Association's Declaration of Helsinki 2013, the work of this article is registered in the Research Registry, and the unique identifying number is: researchregistry5159.The link to the registration page is: https://www.researchregistry.com/browse-the-registry#home/.

**Ethical approval:** Ethical committee approval granted from the Directorate of General Health, Scientific Research Division at the April 23, 2019 with reference number 12032019–2.

### 2.4 Statistical analysis

These data analyzed and correlated to various patient characteristics displayed in terms of frequency, mean, median, and standard deviations. The correlation is done using the two-tailed t-tests, chi-square test (χ2), and Fisher's exact test. In the results of the analyses with 95% confidence interval, values p < 0.05 were considered significant. The statistical calculations were done in the Statistical Package for Social Sciences (SPSS 25:00 IBM: USA).

## 3 Results

The mean age of the patients who were included in the study was 34 years ranged 9–65 years. Most of the patients were on their menstruating circle (88.3%), have regular menstrual (81.3%), and have a history of lactation (75.3%), were non-smokers (93.9%), non-alcoholic (98.4%), not-coffee drinkers (93.7%), not taking OCCP (95.7%), and have a negative family history of breast cancer (98.0%), as presented in Table 1.

**Table 1:** Showing the patients characteristics.

Main category	Subcategories	Frequency	Percentage
AGE; m (SD) Range: 9-65		34	10.843
Menstrual status	Menstruating Menopausal	393 52	88.3 11.7
Menstrual cycles	Regular Irregular	362 83	81.3 18.7
Lactation status	Lactating Non-lactating History of lactation	17 93 335	3.8 20.9 75.3
Nipple discharge	Yes No	84 361	18.87 81.13
Smoking history	Smoker Non-smoker	27 418	6.1 93.9
Alcohol consumption	Alcoholic Non-alcoholic	7 438	1.6 98.4
Coffee consumption	Coffee drinking No coffee drinking	28 417	6.3 93.7
OCCP	Taking OCCP No OCCP	19 426	4.3 95.7
Family history of breast cancer	Positive family history of breast cancer Negative family history of breast cancer	9 436	2.0 98.0

**Fig. 1:**
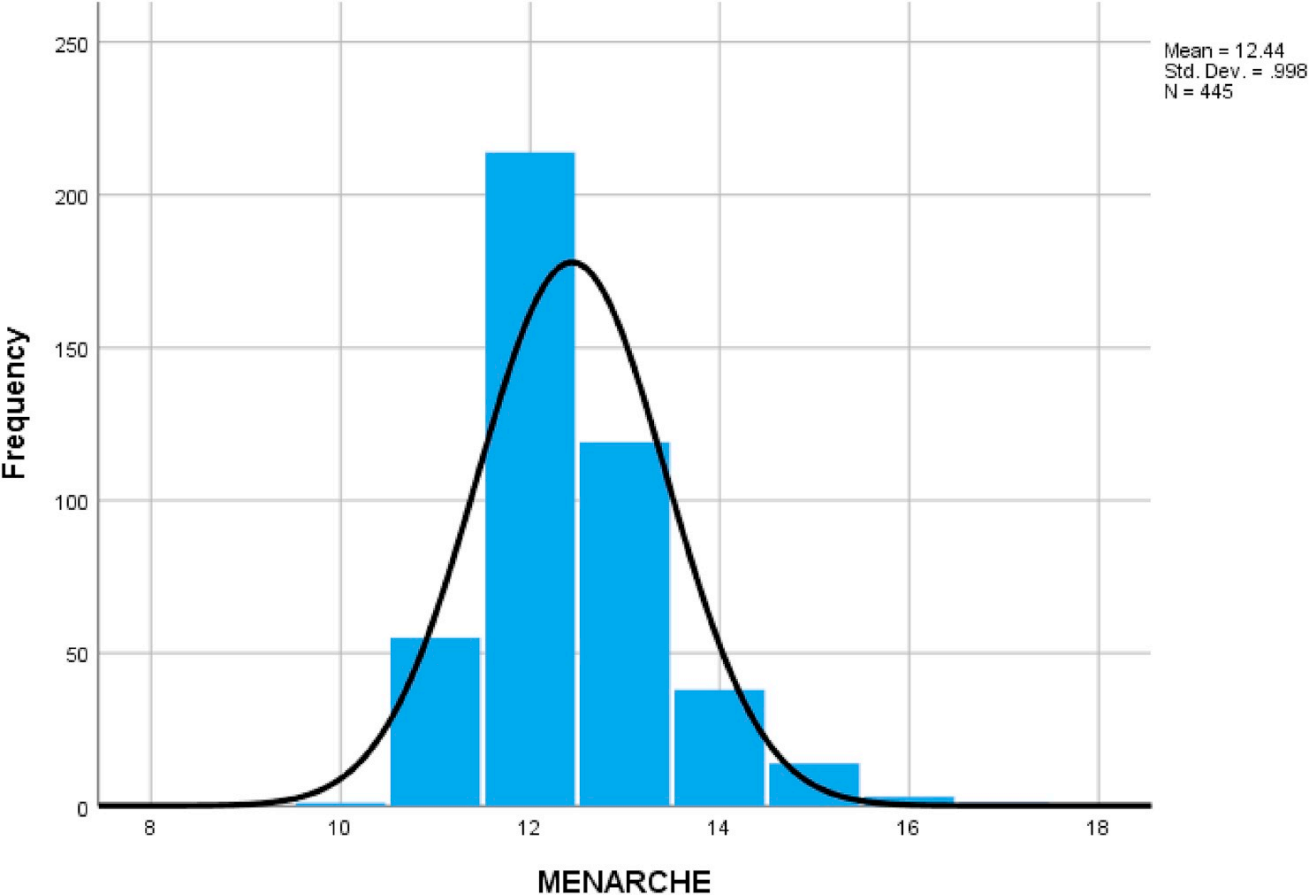
A histogram showing the age of menarche in our patients.

The vast majority of the patients included in this study had menarche at 12 years of age, and the maximum age for menarche was 17 years among our patients, Fig. 1.

The majority of our patients have noncyclical pain (70.1%) and mostly felt in the left breast (45.8%), see Table 2.

**Table 2:** Showing the analysis of pain.

Main category	Subcategories	Frequency	Percentage
Type of pain	Cyclical	133	29.9
	Non-cyclical	312	70.1
Site of pain	Right breast	134	30.1
	Left breast	204	45.8
	Bilateral	107	24.0
Pain in other sites (neck and shoulder)	Yes	18	4.0
	No	427	96.0

The mastalgia showed significant correlation with smoking status (P < 0.001), history of oral contraceptive pills uses (P 0.009), and family history with breast cancer (P < 0.001) while there was no association with other factors, Table 3.

**Table 3:** Showing the relation of mastalgia to different types of patient characteristic features.

	Standardized Coefficients^a^	t	P-Value
	Beta		
Age	0.109	1.118	0.264
Occupation	−0.018	−0.293	0.770
Weight	−0.096	−1.631	0.104
Menarche	−0.007	−0.142	0.887
Menstrual status	0.028	0.361	0.718
Menstrual cycles	−0.056	−1.021	0.308
Marital status	−0.062	−0.799	0.425
Age at 1st pregnancy	−0.014	−0.217	0.828
Number of children	−0.055	−0.720	0.472
Lactation status	−0.011	−0.171	0.864
Smoking status	−0.341	−5.523	**<0.001**
Alcohol consumption	0.032	0.554	0.580
Coffee consumption	0.038	0.699	0.485
History of OCCP	0.162	2.638	**0.009**
Family history of breast cancer	−0.181	−3.543	**<0.001**
Site of pain	−0.045	−0.866	0.387
Nipple discharge	0.029	0.559	0.577
Previous breast surgery	−0.022	−0.425	0.671
Pain in other sites	0.031	0.543	0.587

Abbreviations: OCCP: Oral contraceptive pills.

The bold numbers show the predictors.

a Dependent variable: pain.

Analysis of the type of mastalgia, whether cyclical or noncyclical further analyzed and correlated with the patient factors. It showed a significant correlation with the age of the first pregnancy and the site of the pain, Table 4.

**Table 4:** Showing the relation of the type of mastalgia for different kinds of patient characteristic features.

	Standardized Coefficients^a^	t	Sig.
	Beta		
Age	.183	2.066	.040
Weight	.057	1.096	.274
Menarche	.018	.382	.703
Menstrual status	-.050	-.715	.475
Menstrual cycles	-.085	−1.703	.089
Age at 1st pregnancy	.175	3.524	**.001**
Number of children	.151	2.340	.020
Lactation status	-.016	-.294	.769
Smoking status	.047	.851	.395
Alcohol consumption	-.029	-.561	.575
Coffee consumption	-.031	-.614	.539
History of OCCP	.015	.271	.787
Family history of breast cancer	-.061	−1.311	.191
Site of pain	-.162	−3.378	**.001**
Location of pain	.082	1.696	.091
Nipple discharge	.012	.245	.807
Previous breast surgery	.030	.620	.535
Pain in other sites	-.033	-.630	.529

Abbreviations: OCCP: Oral contraceptive pills.

The bold numbers show the predictors.

a Dependent variable: Type of pain.

The majority of our patients were assessed using the ultrasound, when the diagnosis was not conclusive patients were sent for MRI, mammography was performed for a small number of our patients because most of them were young, Fig. 3.

**Fig. 2:**
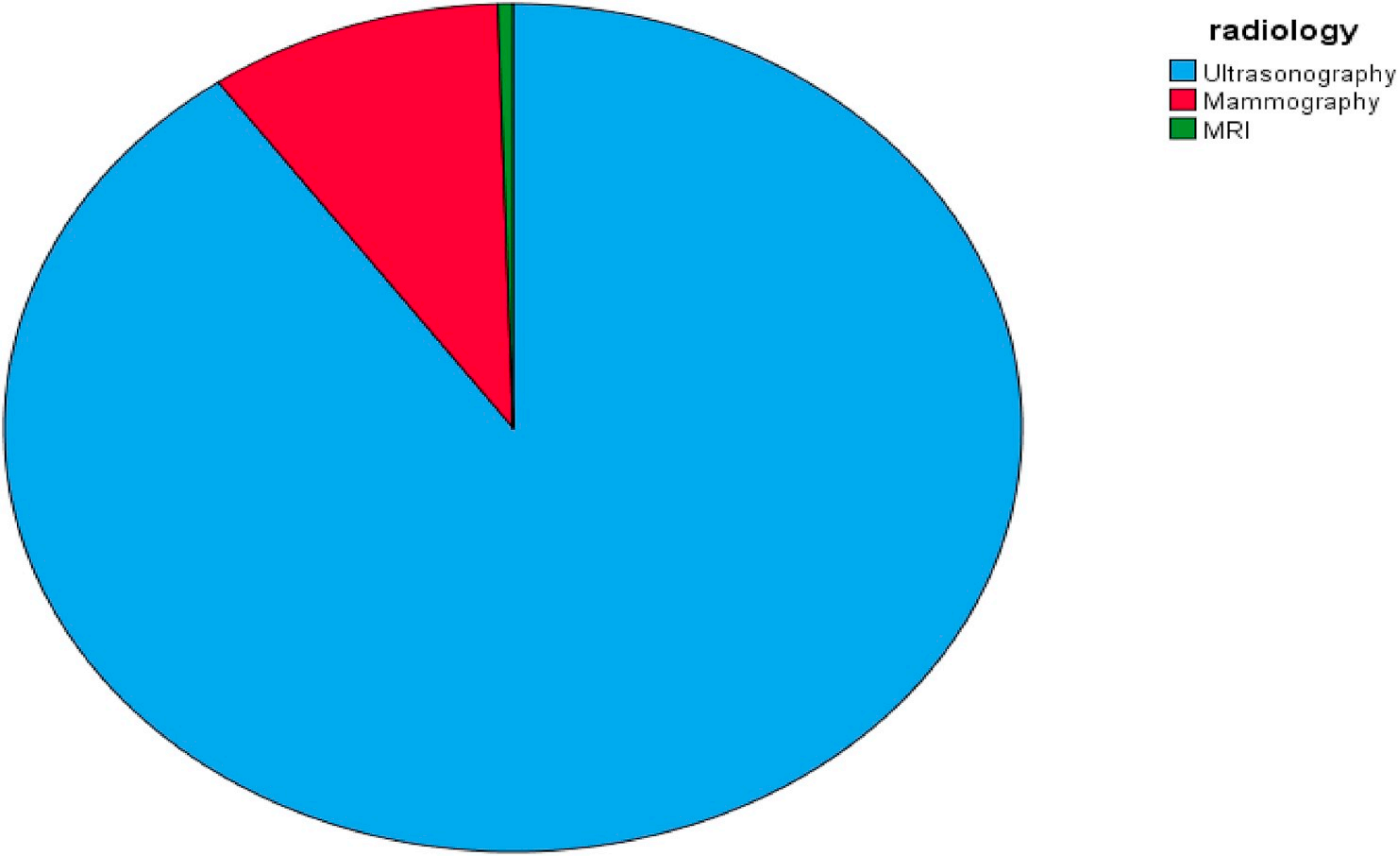
A simple pie chart showing the imaging modalities performed for our patients.

The mean age of age of first pregnancy in our patients was 20.9 years, which is considered young when compared with the world data, Fig. 2.

**Fig. 3:**
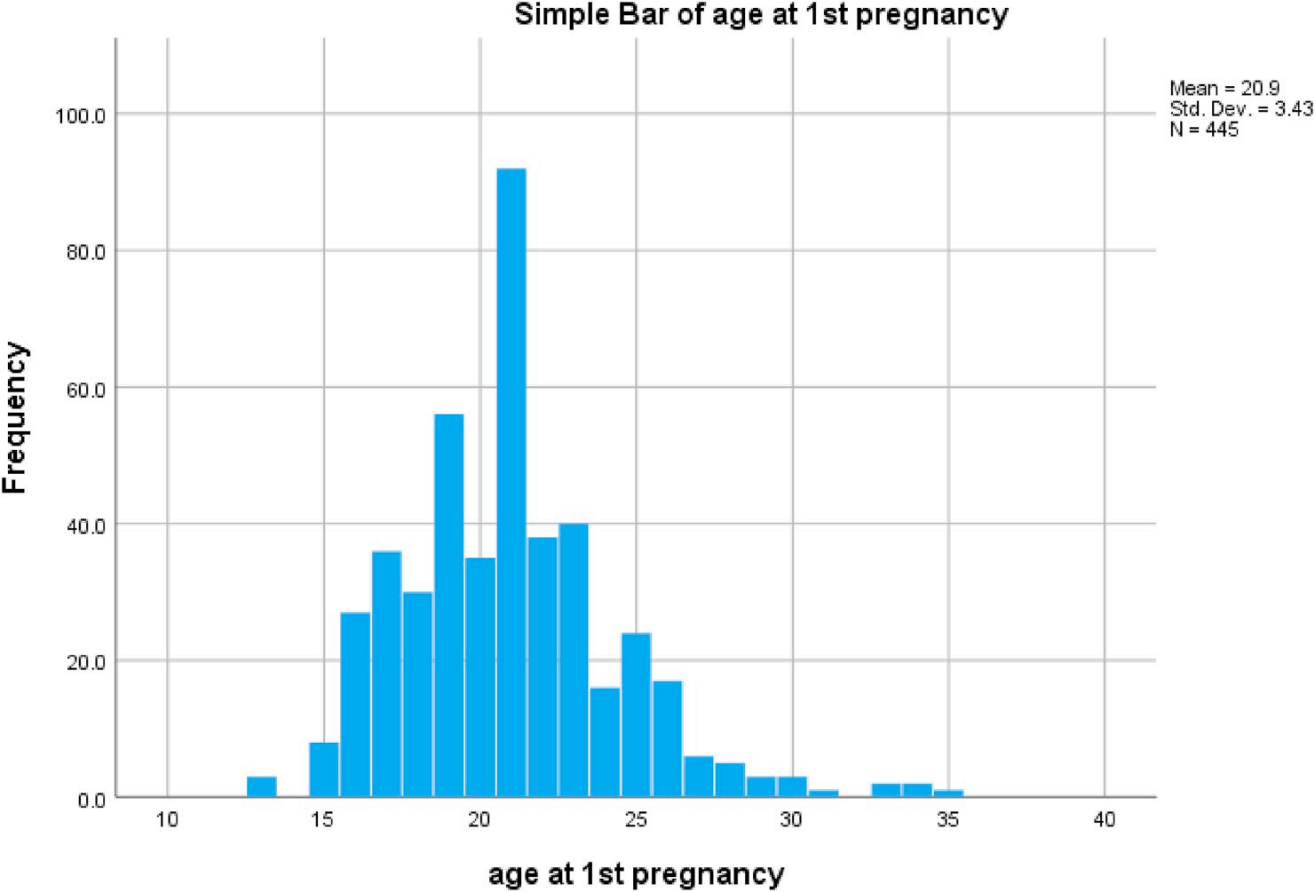
A simple bas chart showing the ages of the first pregnancy among our patients.

## 4 Discussion

There was a lot of debate regarding whether the type of the pain in mastalgia is a psychoneurotic pain or have a pathological base, some studied concluded that mastalgia should not be more regarded as a psychoneurotic pain because the majority of such patients have either a physiological or a pathological cause [12].

Stress and smoking were negatively associated with mastalgia among our patients. Patients with a positive family history of breast cancer had a higher percentage of noncyclical mastalgia among them, this could be explained by the fear and stress of developing breast cancer, for this reason, and after excluding breast, cancer reassurance should be the first line of management, and it is an effective line in alleviating the breast pain. Studies showed a similar correlation with the same factors [3,13,14].

In some patients, the pain may settle spontaneously without any form of treatment [7].

A low-fat diet, high carbohydrate, regular exercise, weight reduction, well-fitting bra, and breast support are also included as a first line treatment; some drugs can be used as first-line medications including topical and oral non-steroidal anti-inflammatory preparations. Topical non-steroidal anti-inflammatory preparations are mainly effective when mastalgia is localized, but when involving the whole breast, its efficacy is reduced. Wearing sport brassieres during the day time help to prevent the active breast movements during the daily activities helping to decrease the breast pain because the breasts have weak suspensory ligaments [1,7,15].

The majority of our patients were not consuming caffeine-containing drinks, which suggest that caffeine intake is not linked to the causation of mastalgia. So women should be no more advised to reduce caffeine intake as one of the treatment lines of mastalgia [15].

A lot of data are available in the literature about the effectiveness of the evening primrose oil in the treatment of mastalgia, but no sufficient evidence is present about the effectiveness of such treatment in the majority of the patients. Vitamin E preparations are also of low value in the treatment of mastalgia in most patients but may be of value in women with cyclical mastalgia who are taking hormonal therapy as well [15,16].

If the first line therapy is not effective, other modalities of treatments are tried like the hormonal therapy, but such treatment is only indicated in the refractory cases. This hormonal therapy may include anti-prolactin drugs such as bromocriptine, antiestrogens such as tamoxifen and androgen agonists such as danazol. This hormonal therapy is usually given to patients with severe mastalgia, which is not responding to other modalities of treatment. Most patients tend to prefer bromocriptine because of lower side effects and a significant reduction in the level of prolactin. Both cyclical and non-cyclical mastalgia have an approximate response rate [1,17,18].

Danazol showed that it an effective drug in the management of both cyclical and non-cyclical mastalgia [2].

Some newer agents may be used, especially in the intractable cases; these agents may include lisuride maleate [7].

Cyclical mastalgia may be considered as an independent risk factor for the development of breast cancer, but further studies are required with solid evidence and further evaluation. In our study, most of the involved patients were menstruating and we didn't find any significant correlation between the menstrual status and mastalgia [19].

Nipple discharge was present in 18.87% of our patients, we didn't find any significant correlation between mastalgia and nipple discharge, some authors correlated nipple discharge with mastalgia especially the non-cyclical one [20].

In 4% of the patients in our study, the pain had musculoskeletal origin being referred either from the shoulder or from the neck, in these patients the pain intensity was significantly reduced with the analgesics and the local steroid injections. All doctors should be aware of this musculoskeletal type of pain and should be differentiated from mastalgia, especially the noncyclical one for effective management [21].

Intractable mastalgia is seen in about 25% of the noncyclical type and 5% of the cyclical one, in such patients, all the treatment modalities will fail to alleviate the pain. Soma authors had tried a surgical intervention in such groups either with quadrantectomy or even mastectomy with breast implants. Most patients resolved with the mastectomy but complications reported with the use of breast implants, so most authors don't recommend surgery, but when the pain is very severe and intractable for years such treatment can be offered in a very selected group of patients [22].

Goserelin is an effective drug in the management of severe mastalgia, although it has many side effects but provides a significant short term relief of pain [23].

The limitation of this study is that longer term follow up period is required and the population based study may be required. Future studies should include the different types of pain characters, and its association with various physiological breast changes.

## 5 Conclusions

Mastalgia is a very common complain among patients who have breast complains, the most important step in the management is to exclude breast cancer. The management should include a systematic approach starting from the detailed history and breast examination, imaging assessment and in some patients histopathological evaluation is required. The majority respond to life style modifications and analgesics, hormonal therapy may be required in some patients.

## Ethical approval

NA.

## Sources of funding

No source of funding other than the authors.

## Author contribution

Study design, data collection and analysis, writing and final approval of the manuscript: Dr Ayad Ahmad Mohammed.

## Research registration unique identifying number (UIN)

Researchregistry5159.

## Trial registry number – ISRCTN

N/A.

## Guarantor

Dr Ayad Ahmad Mohammed.

## Funding

The author was only financial supporter of the study.

## Declaration of competing interest

No conflicts of interest present.
